# Replacement of sugar in the product formulation of “Bomboyson” by jaggery

**DOI:** 10.1002/fsn3.124

**Published:** 2014-05-29

**Authors:** Ghanendra Gartaula, Mahendra Bhattarai

**Affiliations:** Department of Food Technology, GoldenGate International College, Tribhuvan University affiliateKathmandu, Nepal

**Keywords:** Bomboyson, comparison, formulation, jaggery, khoa, traditional

## Abstract

“Bomboyson” is a traditional heat desiccated dairy product of eastern Nepal, prepared using khoa, sugar, and ghee, and has a great commercial potential. This study was carried out to standardize the product and study its nutritive value and storage life for the organized production. The formulation was based on survey and the best product was selected using 9-point hedonic rating scale method. The sugar in the best product was replaced by jaggery, and both were compared with the most popular market product for sensory and nutritional qualities. The products were stored at ambient (25°C) and refrigerated (5°C) temperatures to study the microbiological qualities. Bomboyson prepared from 100 parts khoa, 40 parts sugar, and 20 parts ghee was found to be superior (*P* < 0.05) in terms of overall sensory attributes. The proximate composition of sugar-added bomboyson was found to be 8.98 ± 0.04% moisture, 28.66 ± 1.16% fat, 16.42 ± 0.56% protein, 1.08 ± 0.12% total ash, 44.86 ± 0.86% carbohydrate, and 91.02 ± 0.04% total solids. The proximate composition of control and sugar-added product were same (*P* > 0.05) while both the product had lower contents (*P* < 0.05) of moisture and total ash than the jaggery-added product. The microbiological study confirmed that the products were acceptable throughout the storage period of 28 days at 5°C and 21 days at 25°C.

## Introduction

“Bomboyson” is a traditional dairy product prepared by cooking khoa with ghee and sugar. It is a sweet product, dark brown in color with somewhat caramelized flavor and obtained in a certain rectangular shapes. It is mainly produced in Ilam, an eastern hilly region of Nepal. Some local sweet-meat makers and small dairy industries produce bomboyson in a low scale. It is believed that the region and local people use this sweet as gifts and souvenirs. It has a potential of commercialization because of its peculiar taste and texture.

Khoa, also known as khoya, is a traditional dairy product, manufactured by boiling fresh milk, generally of cow, buffalo, or mixed, in an open pan kept directly on a fire with continuous agitation and scraping to avoid burning and overheating. It is an important base and/or filler material for the preparation of several indigenous sweets of Indian subcontinent such as “peda,” “burfi,” and “kalakand.” A typical khoa prepared from cow's milk contains 25% moisture, 26% fat, 19% protein, 25% lactose, and 3.8% ash (Bansal 2011). Ghee is a clarified butterfat, usually prepared from cow milk, buffalo milk, or mixed milk (Rajorhia 1993). It is a product obtained from milk, cream, or butter from various animal species by means of processes which result in the almost total removal of moisture and solids-not-fat with a minimum of 96% milk fat (IDF 1977). Sugar is the sweetening agent used in bomboyson. It is virtually devoid of vitamins, minerals, and phytochemicals. Jaggery is wholesome as compared to sugar. It contains 65–85% sucrose as compared to 99.5% of that of sugar. The mineral content of jaggery ranges from 0.6% to 1.0% while sugar contains only 0.05% (Singh 1998). The pharmacological activity of jaggery has also been reported (Nayaka et al. 2009). The World Health Organization (WHO) is considering halving the amount of sugar that it recommends people should have in their diet to reduce diet-related noncommunicable diseases, such as obesity and dental caries. WHO's current recommendation, from 2002, is that free sugars should make up less than 10% of total energy intake per day (WHO 2014).

The product has no well-defined procedure and formulation which varies from manufacturer to manufacturer with regard to khoa, amount of sugar, amount of ghee, and extent of desiccation. From a survey based on questionnaire (data not shown), it was known that for every 100 parts of khoa, 20–40 parts of sugar, and 15–25 parts of ghee are used in its manufacture. So bomboyson differs in quality. Many researches have been done on khoa-based products like gundpak, kunda, lal peda, and peda (Acharya and Sapkota 2008; Mahalingaiah et al. 2014; Jha et al. 2012; Londhe et al. 2012). But there is no literature available on the standardization and optimization of bomboyson. No studies have been carried out on its storage life. Hence, a detailed study for the optimization and improvement of the quality was needed. This could help to form a consistent quality and good market demand in the whole country. Hence, the present work was undertaken to optimize the proportion of ingredients and study the storage life of bomboyson so that its production could be organized and commercialized. Furthermore, an attempt was made to replace sugar by jaggery in order to improve its nutritional quality.

## Material and Methods

### Preparation of bomboyson

**Figure 1:**
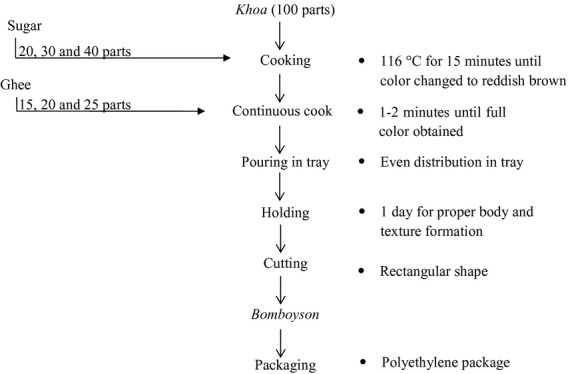
Flow chart for the preparation of bomboyson.

Khoa and ghee were brought from Ilam, Nepal. Sugar and jaggery were bought from the nearby market of Kathmandu. Nine formulations of bomboyson were prepared with 20, 30, and 40 parts sugar and 15, 20, and 25 parts ghee, for every 100 parts khoa. The flow chart for the preparation of bomboyson is given in Figure 1.

The formulations were coded as A, B, C, D, E, F, G, H, and I (Table 1). The prepared samples were subjected to sensory evaluation by semitrained panelists from the Faculty and Research Scholars of Department of Food Technology, GoldenGate International College. Based on the sensory evaluation, the best product (S) was compared to most popular bomboyson (K) of the eastern region (based on local survey, data not shown). Furthermore, sugar in the product S was replaced by jaggery, to yield product J and compared for its sensory, nutritional, and microbiological qualities with S and K.

**Table 1:** Different formulations of bomboyson.

Sample code	Khoa (g)	Sugar (g)	Ghee (g)
A	100	20	15
B	100	20	20
C	100	20	25
D	100	30	15
E	100	30	20
F	100	30	25
G	100	40	15
H	100	40	20
I	100	40	25

### Compositional analysis of the product

Moisture content, crude fat, and crude protein of khoa and bomboyson were determined by methods given by Egan et al. (1991). The ash content and total solid content in khoa and bomboyson were determined following the standard procedure mentioned in AOAC (2005). Carbohydrate content in khoa and bomboyson were determined by the following difference method: Total Carbohydrate (%) = 100 − (Sum of % Moisture, % Fat, % Protein, and % Ash).

### Microbiological analysis

Bomboyson samples K, S, and J were stored under refrigerated (5°C) and at ambient (25°C) conditions in PE pouches for a period of 28 days. Total plate count (TPC), yeast and mold counts, and coliform count were conducted as per standard procedure as mentioned in AOAC (2005). Total plate count was estimated by plating the dilutions using nutrient agar and incubating the Petri plates at 30°C for 72 h, yeast and mold counts were estimated using potato dextrose agar with incubation temperature of 25°C for 48 h, and coliform counts using violet red blue agar and incubation at 30°C for 24 h. The counts were expressed as log colony-forming units (log_10_ cfu) per g of bomboyson.

### Sensory evaluation

Bomboyson was evaluated for color, smell, taste, texture, and overall acceptability on a 9-point hedonic rating scale by semitrained panelists as given by Ranganna (2011). The results obtained were subjected to statistical analysis.

### Data analysis

The scores given by semitrained panelists were analyzed using analysis of variance at 5% level of significance by Genstat software (VSN International Ltd, Hemel Hempstead, UK) and their differences were calculated by using least significant difference method. The comparisons of experimental and control products were also statistically analyzed. All the data were expressed as mean ± standard error of mean calculated from three independent experiments.

## Results and Discussion

The different formulations of bomboyson were subjected to sensory, chemical, and microbiological analysis.

### Sensory analysis

The effect of varying the proportions of sugar and ghee on khoa basis on mean sensory score of A, B, C, D, E, F, G, H, and I are presented in the Table 2. A, B, C, D, E, F, G, H, and I represents the products containing the sugar and ghee in the ratio of 20:15, 20:20, 20:25, 30:15, 30:20, 30:25, 40:15, 40:20, and 40:25 proportions on 100 parts khoa basis, respectively.

**Table 2:** Sensory analysis of Bomboyson.

Formulation	Color	Smell	Taste	Texture	OA
A	5.50^a^	7.13^a^	6.00^a^	5.50^a^	5.88^a^
B	5.00^a^	6.88^a^	6.12^a^	4.88^a^	5.50^a^
C	5.12^a^	7.38^a^	6.38^a^	5.62^a^	6.25^a^
D	6.82^b^	7.00^ab^	7.25^ab^	6.88^b^	7.12^b^
E	5.75^a^	7.5^b^	7.12^ab^	6.38^b^	6.88^b^
F	6.88^b^	7.75^c^	7.50^c^	6.75^b^	7.38^c^
G	7.88^c^	7.13^b^	7.12^ab^	5.38^a^	6.38^bc^
H	8.25^d^	7.63^c^	7.50^c^	7.75^c^	8.38^d^
I	7.75^cd^	7.88^c^	7.88^c^	7.50^c^	7.62^d^

The values are the mean of eight panelists score. The values having different superscript alphabets in a column differ significantly (*P* < 0.05).

The statistical analysis showed that the products vary significantly at 5% level of significance from one another on mean sensory scores.

Results from sensory evaluations of nine preliminary formulations are presented in Table 2. The score of overall acceptance (OA) varied from 5.5 to 8.38. Color, smell, taste, and texture scored 5.00–8.25, 6.88–7.88, 6.00–7.88, and 4.88–7.75, respectively. The OA of the product was concerned. Product H scored the highest which differed significantly (*P* < 0.05) with other products except I (*P* > 0.05). Since product H uses less amount of ghee, it was selected as the best formulation.

In another part of the study, sugar in H (now labeled as S) was replaced by equal amount of jaggery to yield bomboyson J. S and J were compared with K for sensory, chemical, and microbiological analysis.

**Figure 2:**
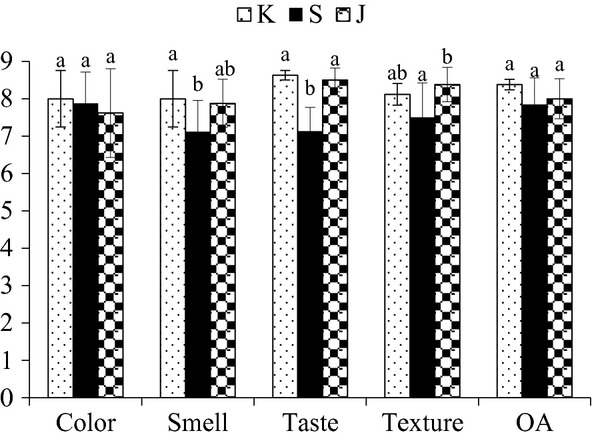
Sensorial comparison of bomboyson. K, S, and J represents control, sugar-added, and jaggery-added bomboyson (*n* = 3). Different alphabets are used to denote significantly different products, for each attribute.

The comparison of sensory analysis of K, S, and J is given in Figure 2. The products differed significantly in terms of smell, taste, and texture. The scores of color, smell, taste, texture, and OA ranged from 7.62 to 8.00, 7.12 to 8.00, 7.13 to 8.63, 7.5 to 8.38, and 7.85 to 8.38, respectively. The three products did not differ significantly (*P* > 0.05) in color appearance. K and S differed (*P* < 0.05) with each other in terms of smell, but were similar (*P* > 0.05) with J. It might be due to the use of jaggery in J. Products K and J were similar (*P* > 0.05) in taste while both of them differed significantly with S. The texture of K was same with that of S and J, but S and J differed (*P* < 0.05). The three products K, S, and J did not differ significantly in terms of OA. As the OA of the product is concerned, it can be inferred that the replacement of jaggery did not alter the OA of the product.

### Chemical analysis

The mean values of chemical composition of control product and experimental products are shown in the Table 3. The statistical analysis of the three products showed that there was no significant difference at 5% level of significance between K and S, while there were significant differences (*P* < 0.05) in moisture content, total ash, carbohydrate, and total solid content with J. The variation in moisture content and total solids might be due to the extent of desiccation during cooking. The increase in ash content in J could be attributed to the high mineral content in jaggery as compared to sugar (Singh 1998).

**Table 3:** Comparison of compositions.

Composition	K	S	J
Moisture	8.49 ± 0.02^a^	8.98 ± 0.04^a^	12.33 ± 0.48^b^
Fat	28 ± 0.19^a^	28.66 ± 1.16^a^	28.88 ± 0.17^a^
Protein	16.8 ± 0.99^a^	16.42 ± 0.56^a^	16.2 ± 0.29^a^
Total ash	1.16 ± 0.02^a^	1.08 ± 0.12^a^	2.17 ± 0.67^b^
Carbohydrate	45.55 ± 0.85^a^	44.86 ± 0.86^a^	40.42 ± 0.79^b^
Total Solid	91.51 ± 0.03^a^	91.02 ± 0.04^a^	87.67 ± 0.48^b^

K, S, and J represents control, sugar-added, and jaggery-added bomboyson (*n* = 3). Different superscript alphabets are used to denote significantly different products (*P* < 0.05) for each attribute.

### Microbiological analysis

**Figure 3:**
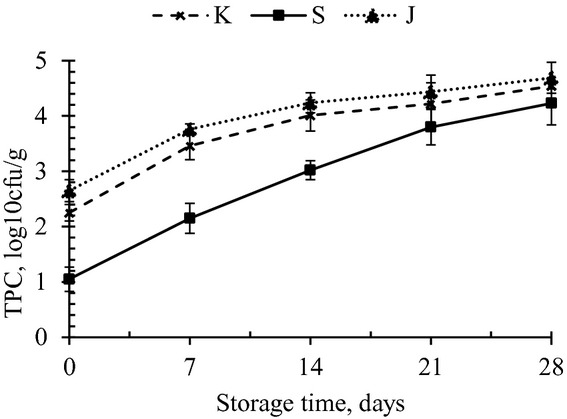
Total plate count of bomboyson stored for 28 days at refrigerated temperature.

**Figure 4:**
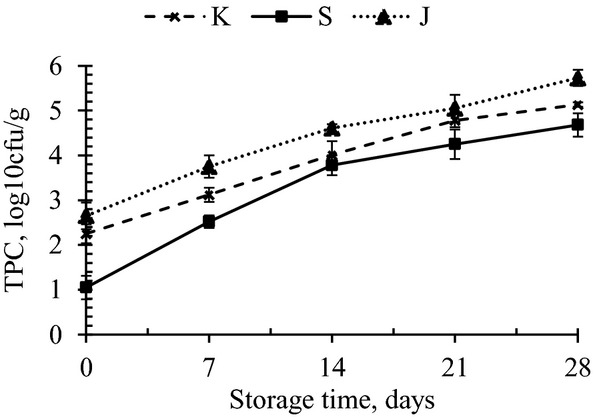
Total plate count of bomboyson stored for 28 days at room temperatuure. K, S, and J represents control, sugar-added, and jaggery-added bomboyson (*n* = 3).

Bomboyson samples were analyzed at an interval of 7 days of storage at ambient and refrigerated conditions for microbial changes. The initial population of TPC in K, S, and J were 2.25, 1.05, and 2.65 log_10_ cfu/g, respectively. Yeast and mold colonies were seen in J in the incubation period of 28 days. Coliforms were not detected in the samples throughout the study period of 28 days. The TPC of bomboyson stored at refrigerated and ambient conditions are presented in Figures 3, 4, respectively.

Nepal Government has microbial limits only for limited milk products. The critical limits of TPC in infant milk food, whole milk powder, and skimmed milk powder are 4.60, 4.69, and 4.60 log_10_ cfu/g, respectively (DFTQC 2012). FSSAI (2011) has set the maximum limits for TPC, coliform, and yeast and mold counts in khoa as 5, 1.95, and 2 log_10_ cfu/g, respectively.

At refrigeration temperature, the TPC counts for K, S, and J rose to 4.54, 4.23, and 4.69 log_10_ cfu/g, respectively, on 28 days of storage, while at room temperature, the counts were 5.13, 4.68, and 5.73 log_10_ cfu/g, respectively. The yeast and mold count of three products showed no colonies up to 21 days of storage. While on 28 days, J showed 3.6 log_10_ cfu/g at room temperature and 3.4 log_10_ cfu/g at refrigeration temperature. Hence, based on the available microbiological standards, it can be reported that the products were acceptable throughout the storage period of 28 days at refrigerated temperature and 21 days at room temperature. Lal peda samples stored at 4 and 37°C were acceptable up to 31 and 9 days, respectively, on the basis of textural and sensory attributes (Jha et al. 2012). In a study by Jha et al. (2013), when lal peda samples were stored under air as packaging atmosphere of 10°C, microbial activity reached the critical limits (5.31 log_10_ cfu/g) within 30–40 days. Kunda, a heat desiccated dairy product, was acceptable throughout the storage period of 42 days at 30°C and 90 days at 5°C (Mahalingaiah et al. 2014). The shelf life could be extended by storage at lower temperatures.

## Conclusions

Bomboyson prepared by the incorporation of 100 parts khoa, 40 parts sugar, and 20 parts ghee gave the best result in terms of sensory analysis. Replacing the sugar content by jaggery did not alter the OA of the product and there is increase in the mineral content of the product. The product could be stored safely up to 28 days at 5°C and 21 days at 25°C.
